# Caffeine and Modafinil Ameliorate the Neuroinflammation and Anxious Behavior in Rats during Sleep Deprivation by Inhibiting the Microglia Activation

**DOI:** 10.3389/fncel.2018.00049

**Published:** 2018-02-28

**Authors:** Meetu Wadhwa, Garima Chauhan, Koustav Roy, Surajit Sahu, Satyanarayan Deep, Vishal Jain, Krishna Kishore, Koushik Ray, Lalan Thakur, Usha Panjwani

**Affiliations:** Defence Institute of Physiology & Allied Sciences (DIPAS), Defence Research and Development Organization (DRDO), New Delhi, India

**Keywords:** sleep deprivation, mood changes, microglia, cytokines, neuroinflammation, caffeine, modafinil

## Abstract

**Background**: Sleep deprivation (SD) plagues modern society due to the professional demands. It prevails in patients with mood and neuroinflammatory disorders. Although growing evidence suggests the improvement in the cognitive performance by psychostimulants during sleep-deprived conditions, the impending involved mechanism is rarely studied. Thus, we hypothesized that mood and inflammatory changes might be due to the glial cells activation induced modulation of the inflammatory cytokines during SD, which could be improved by administering psychostimulants. The present study evaluated the role of caffeine/modafinil on SD-induced behavioral and inflammatory consequences.

**Methods**: Adult male Sprague-Dawley rats were sleep deprived for 48 h using automated SD apparatus. Caffeine (60 mg/kg/day) or modafinil (100 mg/kg/day) were administered orally to rats once every day during SD. Rats were subjected to anxious and depressive behavioral evaluation after SD. Subsequently, blood and brain were collected for biochemical, immunohistochemical and molecular studies.

**Results**: Sleep deprived rats presented an increased number of entries and time spent in closed arms in elevated plus maze test and decreased total distance traveled in the open field (OF) test. Caffeine/modafinil treatment significantly improved these anxious consequences. However, we did not observe substantial changes in immobility and anhedonia in sleep-deprived rats. Caffeine/modafinil significantly down-regulated the pro- and up-regulated the anti-inflammatory cytokine mRNA and protein expression in the hippocampus during SD. Similar outcomes were observed in blood plasma cytokine levels. Caffeine/modafinil treatment significantly decreased the microglial immunoreactivity in DG, CA1 and CA3 regions of the hippocampus during SD, however, no significant increase in immunoreactivity of astrocytes was observed. Sholl analysis signified the improvement in the morphological alterations of astrocytes and microglia after caffeine/modafinil administration during SD. Stereological analysis demonstrated a significant improvement in the number of ionized calcium binding adapter molecule I (Iba-1) positive cells (different states) in different regions of the hippocampus after caffeine or modafinil treatment during SD without showing any significant change in total microglial cell number. Eventually, the correlation analysis displayed a positive relationship between anxiety, pro-inflammatory cytokines and activated microglial cell count during SD.

**Conclusion**: The present study suggests the role of caffeine or modafinil in the amelioration of SD-induced inflammatory response and anxious behavior in rats.

**Highlights**

- SD induced mood alterations in rats.

- Glial cells activated in association with the changes in the inflammatory cytokines.

- Caffeine or modafinil improved the mood and restored inflammatory changes during SD.

- SD-induced anxious behavior correlated with the inflammatory consequences.

## Introduction

Insufficient sleep is one of the most common and significant health problem worldwide associated with the immune system modulation (Dworak et al., 2011) and mood decline (Babson et al., 2010; Alkadhi et al., 2013). Documented evidence support the high prevalence of anxiety and depression with an associated link to inflammation in several pathological conditions like rheumatoid arthritis, kidney disease and bowel disease conditions (Kang et al., 2011). Cytokines play a crucial role in inflammation, neurobehavioral and emotional deficits. During the inflammatory challenge, microglial cells get activated and affect the release of cytokines (pro-inflammatory cytokines increase and anti-inflammatory cytokines decrease), often coinciding with behavioral manifestations (Kang et al., 2014; Wohleb et al., 2014b).

There are growing lines of evidence showing bi-directional communication between the sleep and immune system. Sleep influences the immune system and vice versa (Zielinski and Krueger, 2011). The pro-inflammatory cytokines such as interleukin-1β (IL-1β), TNF-α, IL-6 are found to be increased upon sleep deprivation (SD) in humans as well as experimental animals. Glial cells comprise the innate immune cells of the brain. Once activated, these cells imbalance the cytokine levels leading to behavioral abnormalities. However, their role under sleep-deprived conditions is remained unclear (Wisor et al., 2011; Alkadhi et al., 2013). It had reported that insufficient sleep decreases the mental performance and increases the risk of immune dysfunctions (Carey et al., 2011). Pro-inflammatory cytokines are associated with SD and mood disorders, however, the underlying mechanism is poorly understood (Rönnbäck and Hansson, 2004; Abelaira et al., 2013; Hong et al., 2016).

Caffeine and modafinil are widely consumed psychoactive drugs in the world showing beneficial effects on cognitive performance. Caffeine acts as a non-selective adenosine antagonist showing stimulant activity and prevents the deterioration of the cognitive performance (Daly, 2007; Nehlig, 2010; Sanday et al., 2013; Cappelletti et al., 2015). Concurrently, modafinil acts as a cognitive enhancer after directly binding to dopamine transporter and elevates the level of serotonin (Minzenberg and Carter, 2008; Rasetti et al., 2010). Caffeine or modafinil are thought to improve the mood (Cunha and Agostinho, 2010; Boele et al., 2013). Available literature suggests the dose-dependent effect of caffeine on mood showing an anxiolytic effect at low or moderate doses and anxiogenic effects at higher doses (Rusconi et al., 2014; Yamada et al., 2014).

Human and animal studies have shown improved memory performance after SD upon taking caffeine and modafinil (McGaugh et al., 2009; Cunha and Agostinho, 2010). Caffeine/modafinil administration prevents the neuroinflammation mediating memory disturbance in animal models of Alzheimer’s, Parkinson’s, stress, diabetes, convulsions, or alcohol-induced amnesia (Brothers et al., 2010; Raineri et al., 2012; Gyoneva et al., 2014a). However, neither the detailed mechanisms underlying neuroinflammation mediated emotional regulation during SD nor the effectiveness of the psychostimulants agents has been established yet. Therefore, we selected the two well-known psychostimulants viz., caffeine and modafinil; to assess the mood changes, astrocytes and microglial cells investigation along with the inflammatory cytokine levels during SD. Additionally, the predicted mechanism was investigated by the correlation analysis between the behavioral and inflammatory test parameters.

## Materials and Methods

### Animals

Adult male Sprague-Dawley rats of 6–8 weeks old and approximately 220 ± 10 g body weight were used for the present study. Rats were housed in the clean cages made up of plexiglass material in the animal house at a temperature of 25 ± 2°C and humidity of 55 ± 2% RH with 12 h light and dark cycles with food and water *ad libitum*. All the experimental protocols were approved by the Institutional Animal Ethics Committee (IAEC, IAEC/DIPAS/2015-19) of Government of India, in accordance with the Committee for the Purpose of Control and Supervision of Experiments on Animals (CPCSEA) guidelines. Animal handling was done regularly to make them habituate to the experimenter. Experiments were conducted during the light period of the day. All efforts were done to minimize the number of rats used and to avoid unnecessary pain to the animal.

### Chemicals and Reagents

Analytical grade chemicals were procured from Sigma Chemicals (Sigma-Aldrich, St. Louis, MO, USA) unless otherwise mentioned. The Enzyme-Linked Immunosorbent Assay (ELISA) kits were purchased from BD Biosciences Laboratory Ltd. (USA) and R and D Systems, Minneapolis, MN, USA. Antibodies (primary and secondary) were procured from Sigma-Aldrich, St. Louis, MO, USA, Abcam, Cambridge, MA, USA and Millipore, CA, USA.

### Experimental Design

**Figure 1:**
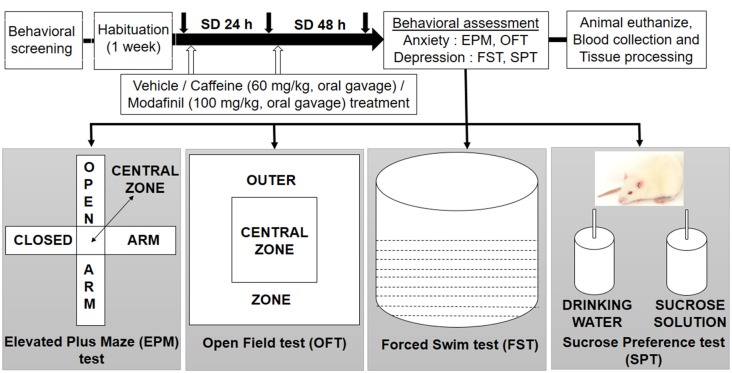
Schematic design of the experiment.

Initially, behavioral screening of rats was done, in which the body weight, food intake, aggressiveness and stereotype behavior were evaluated. This was done to ensure that rats were not suffering from any impairment, after that the animals were divided randomly into different groups: cage control with vehicle treatment (CC+Veh); cage control with caffeine treatment (CC+Caf); cage control with modafinil treatment (CC+Mod); sleep deprived for 48 h with vehicle treatment (SD+Veh); sleep deprived for 48 h and caffeine treatment (SD+Caf); sleep deprived for 48 h and modafinil treatment (SD+Mod). The rats underwent the vehicle and drugs treatment during the control and sleep deprived conditions for 48 h. Each group had five rats, and the behavioral study took place between 8:00 AM and 11:00 AM. We used a different set of rats in each behavioral paradigm. Animals were euthanized immediately after the behavioral test during the light period of the day and evaluated for the biochemical and immunohistochemical analyses. The schematic experimental design of the present study is shown in Figure 1.

### Sleep Deprivation Procedure

Briefly, the male Sprague-Dawley rats were sleep deprived for 48 h in the automated SD apparatus according to the well-established SD protocol of our lab (Wadhwa et al., 2015; Chauhan et al., 2016). The exposure paradigm made the rats awake on providing the SD stimulus such as sound, light, and vibration. There was a proper provision of ventilation, food and water during the SD exposure. The control rats were kept under controlled conditions (temperature, humidity and light) in the animal house facility.

### Drug Administration

Caffeine (Sigma-Aldrich, St. Louis, MO, USA, 60 mg/kg/day, dissolved in physiological saline, administered orally) and modafinil (Modalert, Sun Pharma, India; 100 mg/kg/day, suspended in physiological saline, treated orally) dose were based upon our previous study (Sahu et al., 2013; Wadhwa et al., 2015), however, the tested doses of caffeine were higher and did not mimic habitual caffeine consumption. Caffeine or modafinil was given to the rats as an oral gavage, once a day in the morning time, during 48 h of SD.

### Body Weight and Food Intake

To evaluate the physiological consequences, we monitored the body weight and food intake of both control and experimental rats regularly in the morning (1 week before the initiation of the experiments). The food was maintained at a constant amount (150 g) per animal, and every morning (8:00–9:00 AM), the remaining food was measured. At the same time, the body weight of each animal was noted down.

### Behavioral Testing

We utilized a battery of behavioral tests that measure anxiety and depressive-like behavior including the open field (OF, locomotor activity, exploratory behavior), elevated plus maze (EPM, locomotor activity, exploratory behavior, anxiety), forced swim test (FST, behavioral “despair”) and sucrose preference test (SPT, anhedonia). Each behavioral analysis was carried out with 15 rats in each experimental group.

#### Elevated Plus Maze Apparatus and Test Procedure

The EPM test is used to assess the level of anxiety in rodents. The EPM test apparatus consisted of plus shape design with four arms (two open and two closed, perpendicular to each other) having an open roof. The apparatus was about 40–70 cm elevated from the floor. Briefly, the rat was placed at the junction of the open and closed arms, facing the open arm opposite to the experimenter for 5 min. At the end of the test, rats were removed from the apparatus and placed back to their home cage. The test apparatus was properly cleaned with alcohol and dried with cotton before testing another rat. An overhead camera in association with ANY-maze software (Stoelting Co, Wood Dale, IL, USA) was properly arranged for the tracking and automatically recording the number of entries and time spent by the rat in the open and closed arms. The anxious behavior was evaluated by calculating the proportion of the time spent (time spent into the open arms divided by the total time spent in the open/closed arms) and the proportion of the number of entries (entries into the open arms divided by the total entries into the open/closed arms).

##### Open field apparatus and test procedure

OF test is a well-known method to assess the spontaneous locomotor activity in rats. The OF maze was divided into two zones: central and peripheral zone, using the square drawn on the maze. The apparatus consisted of a rectangular area of 81 × 81 cm surrounded by a 28 cm high wall. The field was lit with white light (23W) fixed 100 cm above the field. The rat was placed in the center of the OF, and its activity during the subsequent 5 min was recorded using ANY-Maze tracking software (Stoelting Co, Wood Dale, IL, USA). The test apparatus was properly cleaned with alcohol and dried with cotton before testing another rat to exclude any cues and smell.

##### Forced swim apparatus and test procedure

The FST was used to assess the depression in rodents. It is based on the assumption that an animal will try to escape, if the rat fails, the animal eventually stops trying and gives up. The FST apparatus is a vertical plexiglas cylinder (40 cm high; 20 cm in diameter) filled with 30 cm deep water (24–30°C). Briefly, the rat was placed in a cylindrical container of water from which it cannot escape, for 5 min. The rat was properly dried after removal from the water with a clean towel. The water was replaced regularly with fresh water to avoid the accumulation of the urine and fecal material. ANY-maze software (Stoelting Co, Wood Dale, IL, USA) was used to determine the test parameters.

##### Sucrose preference test procedure

The anhedonia, indicator of depression, means the lack of interest in rewarding stimuli. In this task, we assessed the animal interest in seeking out a sweet, rewarding drink in plain drinking water. This test was carried out in the animal’s home cage. Briefly, rats were initially habituated to the presence of two bottles; one containing 2% sucrose solution and another drinking water for 2 days in their home cage. During this phase, rats had the free access to both bottles. The intake of normal drinking water and sucrose solution was measured daily, and the positions of bottles were regularly interchanged to reduce biases. On the completion of 48 h SD, the rats were presented the same two bottles (one containing water and another containing sucrose solution) and measured the intake of water and sucrose solution. Sucrose preference index was calculated as a ratio of the volume of sucrose intake over the total volume of fluid intake.

### Blood Collection and Tissue Processing

After the scheduled period of SD exposure and the behavioral assessment, the blood was collected from left ventricle under anesthesia (ketamine 80 mg/kg-xylazine 20 mg/kg) in the vacutainer tube containing the sodium heparin as an anticoagulant. The blood was centrifuged at 3500 rpm at 4°C for 10–15 min, and plasma was separated. The rats were euthanized, and the brain was extracted out immediately. The hippocampi were isolated, washed with cold 0.1 M phosphate buffer saline (PBS) solution. The hippocampus was snap frozen in liquid nitrogen and then stored at −80°C until the time of analysis. Later, the samples were homogenized with the help of Polytron homogenizer (Remi Pvt. Limited) with 1× PBS and protease inhibitor cocktail containing inhibitors with broad specificity for serine, cysteine, acid proteases and aminopeptidases. After homogenization, the solution was centrifuged at 10,000 rpm for 10 min at 4°C, and the supernatant was isolated out for the cytokines assay.

### Cytokine Levels Estimation

The ELISA is a specific and highly sensitive method for the quantification of cytokines. Plasma samples and hippocampal supernatant (100 μl, 1:50 dilution in assay buffer) were assayed for ILs (IL-1β, IL-6, IL-4, IL-10) and TNF-α using commercial ELISA kits. The assays were performed as per the manufacturer’s protocols.

### Evaluation of RNA Expression Levels of Secretory Cytokines in Hippocampus by Real-Time PCR (RT-PCR)

Isolation of the total RNA from hippocampal tissue was done using TRIZOL reagent (Sigma-Aldrich, St. Louis, MO, USA) according to the previously described protocol (Rio et al., 2010). RNA level was quantified using a Nanodrop (Thermo Fisher Scientific, Waltham, MA, USA) by measuring absorbance at 260 and 280 nm. The purity of RNA was checked by denaturing agarose gel electrophoresis and ethidium bromide staining. RNA was reverse-transcribed to cDNA using an RT2 first strand cDNA Synthesis Kit (QIAGEN Sciences, Germantown, MD, USA), according to the manufacturer’s instruction. Relative quantitative analysis of the gene expression of interleukins and TNF for each group was done by employing RT^2^ Profiler inflammatory cytokines and receptor array (QIAGEN Sciences, Germantown, MD, USA) using RT^2^ SYBR^®^ Green qPCR master mix (QIAGEN Sciences, Germantown, MD, USA). The analysis was performed by the comparative 2^−ΔΔCT^ method as previously described. The gene expression analysis was done using software available online at www.sabiosciences.com, after normalization of each gene (Ct) to the housekeeping genes.

### Immunohistochemistry

Transcardial perfusion and fixation were performed using 4% paraformaldehyde (PFA) in 0.1 M PBS (pH = 7.4). Brains were cryosectioned after processing with graded sucrose solution (10%, 20% and 30%) respectively dissolved in PBS (0.1 M, pH 7.4) using cryostat (Leica, Germany). Coronal sections of 30 μm thickness were taken in tissue culture plate and stored at 4°C in sodium azide solution to prevent fungal growth. The sections were processed for immunoreactivity of glial fibrillary acidic protein (GFAP) and ionized calcium binding adapter molecule I (Iba-1) proteins. Briefly, the sections were washed in PBS containing 0.1% Tween-20 or Triton X-100 (PBST) twice for 5 min each, subsequently; the antigen retrieval was done by incubating the sections with sodium citrate buffer for 10–15 min in boiling water bath. Sections were incubated with blocking buffer (5% goat serum for GFAP; 3% bovine serum albumin (BSA) for Iba-1) diluted in PBS for 2 h at room temperature, followed by washing with PBST. Prior to primary antibody labeling of an Iba-1 protein, there was an additional step of permeabilization in which the sections were treated with 0.25% Triton X-100 for 20–30 min followed by PBST washing thrice for 5 min each. The sections were then probed with rabbit anti-GFAP antibody and goat anti-Iba-1 antibody prepared in blocking solution for 40 h at 4°C. Sections were subsequently incubated with biotinylated goat anti-rabbit and rabbit anti-goat antibody for 2 h at room temperature, followed by three washings in PBST (5 min each). Finally, the sections were developed with diaminobenzidine (DAB) tetrahydrochloride solution.

### Imaging and Analysis

The immune-stained sections were observed under Olympus (Melville, NY, USA) BX51TF microscope and images were taken from the DG, CA1 and CA3 regions of the dorsal hippocampus of the brain. We performed sholl analysis for the morphological evaluation of astrocytes and microglial cells. The cell quantification was performed using stereo investigator program. Similarly, immunoreactivity of astrocytes and microglial cells was quantified with the help of ImageJ software.

### Statistical Analysis

All the data are expressed as Means ± SEM. Physiological, behavioral, biochemical, immunohistochemical and molecular data were analyzed by Two-way ANOVA followed by Tukey *post hoc* test with multiple comparisons. Pearson’s correlation test was applied for correlation analysis. Data presented as mean percentage of control value used for graphical representation has been mentioned with graphs. All statistical analysis was done using GraphPad Prism 7.03 Software. The significance level of *p* < 0.05 was considered to be statistically significant.

## Results

### Caffeine or Modafinil Treatment Improved the Physiological Consequences during SD

To assess the physiological changes in rats during SD, their body weight and food intake were recorded. We did not notice changes in body weight gain in the caffeine or modafinil treated control groups compared to vehicle-treated control rats, but a significant decrease in body weight was observed in vehicle-treated sleep-deprived rats as compared to vehicle treated control group rats. Administration of caffeine or modafinil to SD exposed rats significantly improved the body weight as compared to vehicle-treated SD group (*F*_(dFn, dFd); (2,114)_ = 76.28; *p* < 0.0001; Supplementary Figure S1A). However, changes in food intake among the different groups was non-significant (*F*_(2,114)_ = 0.8429; *p* = 0.4331; Supplementary Figure S1B).

### Caffeine/Modafinil Administration Produced Anxiolytic Effect during SD

**Figure 2:**
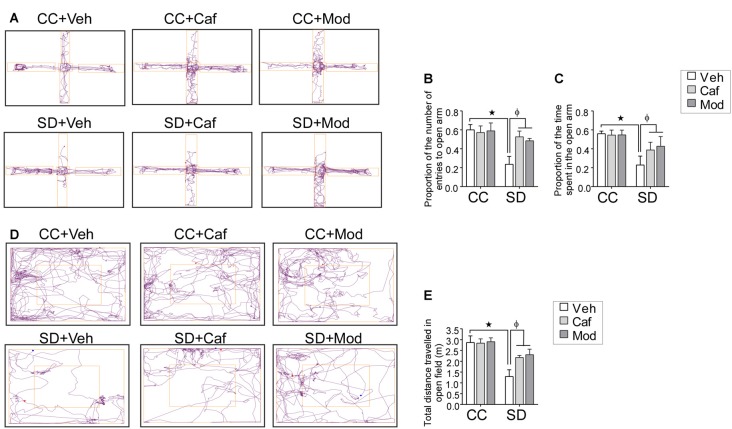
Assessment of the anxious behavior following caffeine/modafinil treatment during sleep deprivation (SD). **(A)** Track plot of the rats during elevated plus maze (EPM) test and the study parameters: **(B)** proportion of the number of entries in the open arms; **(C)** proportion of the time spent in the open arms. **(D)** Track plot of the rats during open field (OF) test; **(E)** total distance traveled in the OF. *p < 0.05 when compared to control treated with vehicle; ^φ^*p* < 0.05 when compared to sleep deprived treated with vehicle. Two way ANOVA followed by Tukey *post hoc* test with multiple comparison was used.

Sleep deprived rats showed anxious behavior while caffeine or modafinil treatment to SD exposed rats improved the anxious behavior of sleep-deprived rats as shown in the track plot of rats during EPM and OF test (Figures 2A,D). The proportion of the number of entries and the proportion of the time spent in the open arms were significantly reduced in sleep-deprived rats treated with vehicle compared to vehicle treated control rats Caffeine or modafinil treatment during SD significantly improved the proportion of the number of entries (*F*_(2,114)_ = 17.4; *p* < 0.0001) and the proportion of the time spent in the open arms (*F*_(2,114)_ = 25.19; *p* < 0.0001) compared to SD exposed rats (Figures 2B,C).

Similarly, in the OF test, the total distance traveled in the OF was significantly reduced in sleep-deprived rats. However, caffeine or modafinil administration significantly improved/increased the total distance traveled in the OF in SD exposed animals compared to sleep-deprived rats (*F*_(2,114)_ = 14.91; *p* = 0.0001; Figure 2E). However, change in vehicle or caffeine, or modafinil treated control rats in EPM and OF test was comparable.

### Caffeine or Modafinil Treatment Recovered the Depressive Behavior during SD

**Figure 3:**
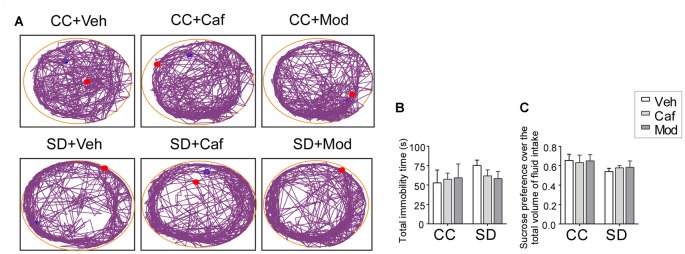
Assessment of the depressive behavior following caffeine/modafinil treatment during SD. **(A)** Track plot of the rats during forced swim test (FST) test; **(B)** total immobility time; **(C)** sucrose preference over the total volume of fluid intake. Two way ANOVA followed by Tukey *post hoc* with multiple comparison test was applied for statistical comparison between groups. Blue dot represented the starting point and the red dot represented the end point of the test.

A non-significant increase in the immobility time was observed in SD exposed animals compared to control animals. Data showed that caffeine/modafinil treatment during SD non-significantly improved the immobility time in SD exposed rats (*F*_(2,54)_ = 1.274; *p* = 0.2881; Figures 3A,B).

Similar to FST, sucrose preference index was non-significantly reduced in rats subjected to SD compared to control, while caffeine/modafinil administration following SD exposure non-significantly improved the sucrose solution intake compared to the sleep-deprived group (*F*_(2,54)_ = 0.6609; *p* = 0.5205; Figure 3C). Also, we did not find a significant difference in the vehicle or caffeine or modafinil-treated control rats.

### Caffeine/Modafinil Administration Maintained the Cytokines Profiling during SD

**Figure 4:**
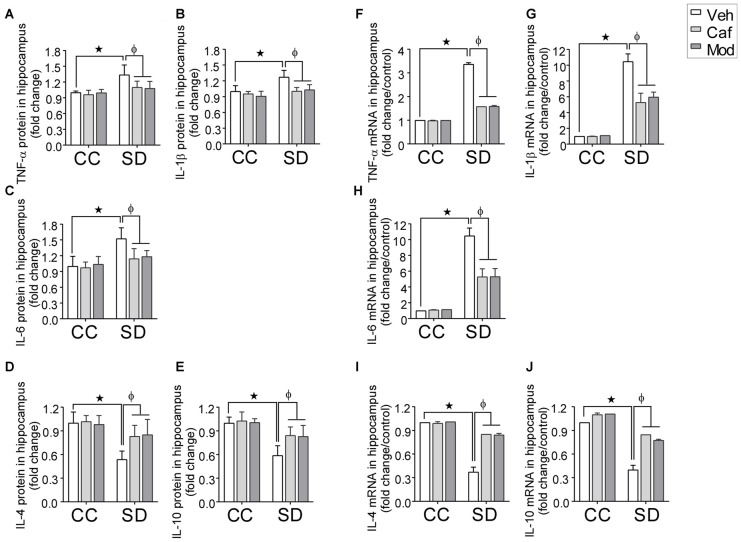
Fold changes in the inflammatory cytokines in hippocampus during caffeine/modafinil administration following SD. The concentration of cytokine levels were measured in picograms per milliliter and expressed as the fold changes in TNF-α **(A,F)**; interleukin-1β (IL-1β) **(B,G)**; IL-6 **(C,H**; pro-inflammatory cytokines), IL-4 **(D,I)**; IL-10 **(E,J**; anti-inflammatory cytokines) in the hippocampus. *p < 0.05 when compared to control treated with vehicle; ^φ^*p* < 0.05 when compared to sleep deprived treated with vehicle. Two way ANOVA followed by Tukey *post hoc* test with multiple comparison were used for the statistical evaluation.

A significant fold increase in the pro-inflammatory cytokines (TNF-α, IL-1β and IL-6) and decrease in the anti-inflammatory cytokines (IL-4 and IL-10) in the hippocampus of rats subjected to SD as compared to control was observed. However, caffeine or modafinil administered during SD significantly decreased the pro-inflammatory: TNF-α (*F*_(2,84)_ = 10.17; *p* = 0.0001; Figure 4A); IL-1β (*F*_(2,84)_ = 3.693; *p* = 0.0290; Figure 4B); IL-6 (*F*_(2,84)_ = 4.168; p = *P* = 0.0188; Figure 4C) and increased the anti-inflammatory cytokines: IL-4 (*F*_(2,84)_ = 22.09; *p* < 0.0001; Figure 4D); IL-10 (*F*_(2,84)_ = 5.933; *p* = 0.0039; Figure 4E) in the hippocampus as compared to vehicle-treated sleep deprived rats.

Subsequently, in plasma, we found a significant fold increase in the pro-inflammatory cytokines and decrease in the anti-inflammatory cytokines insleep deprived rats as compared to control, that were restored by caffeine or modafinil treatment during SD. The respective figures were: TNF-α (*F*_(2,84)_ = 20.32; *p* < 0.0001; Supplementary Figure S2A); IL-1β (*F*_(2,84)_ = 27.67; *p* < 0.0001; Supplementary Figure S2B); IL-6 (*F*_(2,84)_ = 15.39; *p* < 0.0001; Supplementary Figure S2C); IL-4 (*F*_(2,84)_ = 18.52; *p* < 0.0001; Supplementary Figure S2D); IL-10 (*F*_(2,84)_ = 15.2; *p* < 0.0001; Supplementary Figure S2E).

Real time PCR study showed that caffeine or modafinil administration during SD significantly down-regulated the mRNA expression of TNF-α (*F*_(2,12)_ = 2007; *p* < 0.0001; Figure 4F); IL-1β (*F*_(2,12)_ = 27.41, *p* < 0.0001; Figure 4G); IL-6 (*F*_(2,12)_ = 28.36; *p* < 0.0001; Figure 4H), and up-regulated the mRNA expression of IL-4 (*F*_(2,12)_ = 153.9; *p* < 0.0001; Figure 4I), IL-10 (*F*_(2,12)_ = 76.56; *p* < 0.0001; Figure 4J) during SD.

### Caffeine or Modafinil Treatment Down-regulated the Astrocyte and Microglial Cells Immunoreactivity Following SD

**Figure 5:**
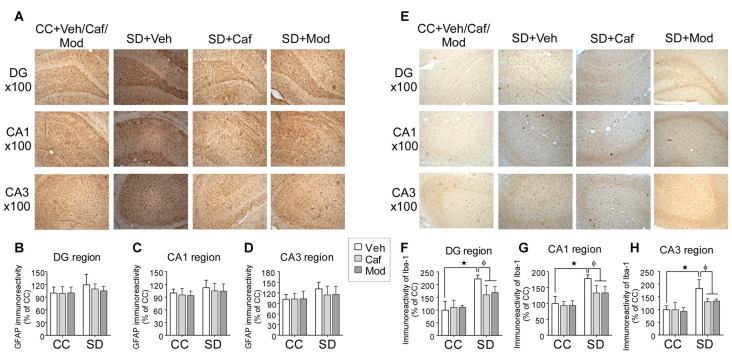
Caffeine or modafinil treatment inhibited the glial cell immunoreactivity in rat hippocampus following SD. **(A)** Representable image of astrocytes expression in DG, CA1 and CA3 regions of the hippocampus. Glial fibrillary acidic protein (GFAP) immunoreactivity quantification in **(B)** DG region; **(C)** CA1 region; **(D)** CA3 region of the hippocampus. **(E)** Representable image of microglial cells expression in DG, CA1 and CA3 regions of the hippocampus. Ionized calcium binding adapter molecule I (Iba-1) cell immunoreactivity quantification in **(F)** DG region; **(G)** CA1 region; **(H)** CA3 region of the hippocampus. *p < 0.05 when compared to control treated with vehicle; ^φ^*p* < 0.05 when compared to sleep deprived treated with vehicle. Two way ANOVA followed by Tukey *post hoc* multiple comparison test was applied for statistical comparison between groups and for the graphical representation, values expressed mean percentage of Control ± SEM.

Astrocytes and microglial cells activation were evaluated by studying the immunohistochemical changes in the expression of GFAP and Iba-1 protein in different regions of the dorsal hippocampus. Changes in the expression of astrocytes among different groups in DG, CA1 and CA3 region of the dorsal hippocampus was shown in Figure 5A. We found no significant increase in the relative mean pixel intensity of GFAP positive cells (astrocytes immunoreactivity) in DG, CA1 and CA3 region of the hippocampus, belonged to SD exposed rats compared to control rats. Caffeine or modafinil treatment non-significantly decrease the astrocytes immunoreactivity in DG (*F*_(2,84)_ = 0.6826; *p* = 0.5081; Figure 5B); CA1 (*F*_(2,84)_ = 0.2293; *p* = 0.7956; Figure 5C), and CA3 (*F*_(2,84)_ = 0.1089; *p* = 0.8970; Figure 5D) region of dorsal hippocampus compared to sleep deprived vehicle-treated rats.

Similarly, the vehicle and drugs treated rats showed changes in the expression of microglial cell in different hippocampal regions as shown in Figure 5E. We found a significant increase in the relative mean pixel intensity of microglial cell in SD exposed rats compared to control rats. Caffeine or modafinil treatment during SD significantly decreased the immunoreactivity of microglial cell in DG (*F*_(2,84)_ = 44.32; *p* < 0.0001; Figure 5F); CA1 (*F*_(2,84)_ = 30.26; *p* < 0.0001; Figure 5G), and CA3 (*F*_(2,84)_ = 25.97; *p* < 0.0001; Figure 5H) region of the hippocampus in comparison with vehicle-treated SD rats. Furthermore, no significant change in the immunoreactivity of GFAP and Iba-1 positive cells was observed following treatment of caffeine or modafinil to control rats compared to vehicle-treated control rats.

### Caffeine or Modafinil Administration Efficiently Improved the Astrocyte and Microglial Cells Morphology Following SD

**Figure 6:**
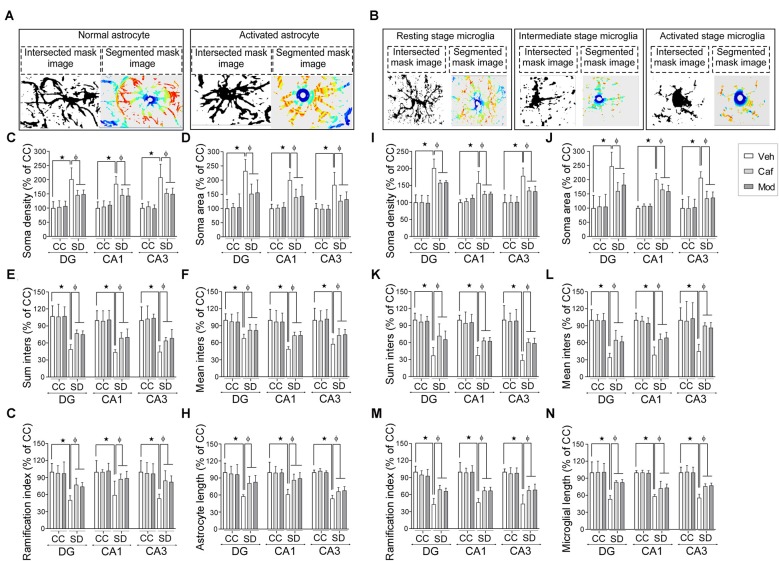
Caffeine or modafinil administration altered the morphology of astrocytes and microglia towards the resting state in rat hippocampus during SD. Representable image (intersection and segmented mask) of **(A)** normal and activated astrocyte; **(B)** resting, intermediate and activated stage microglial cell. Changes in the **(C)** soma density; **(D)** soma area; **(E)** sum inters; **(F)** mean inters; **(G)** ramification index; **(H)** astrocyte length of astrocytes in DG, CA1 and CA3 regions of the hippocampus. Changes in the **(I)** soma density; **(J)** soma area; **(K)** sum inters; **(L)** mean inters; **(M)** ramification index; **(N)** microglial length of microglia cells. *p < 0.05 when compared to control treated with vehicle; ^φ^*p* < 0.05 when compared to sleep deprived treated with vehicle. Two way ANOVA followed by Tukey *post hoc* test with multiple comparison was applied for statistical comparison between groups and for the graphical representation, values expressed mean percentage of Control ± SEM.

Representable intersectional and segmented images of the resting, intermediate and activated stage of astrocyte and the microglial cell was shown in Figures 6A,B. Astrocyte and microglial cells morphology were investigated by the following parameters such as soma density, soma area, sum inters, mean inters, ramification index and glial cell size/length among different groups in DG, CA1 and CA3 region of the dorsal hippocampus.

Administration of caffeine/modafinil significantly improved the SD-induced changes in the morphology of astrocytes in DG, CA1 and CA3 region of the hippocampus (Figures 6C–H; Table 1). Subsequently, microglial cell showed significantly increase in soma density, soma area and a decrease in sum inters, mean inters, ramification index, microglia length following SD while after caffeine or modafinil treatment during SD, the above consequences were significantly improved (Figures 6I–N; Table 1). Statistically non-significant morphological changes were observed in the vehicle and drugs treated control rats hippocampus.

**Table 1:** Astrocyte and microglia morphology during sleep deprivation (SD) and caffeine/modafinil administration.

	*F*_(dFn,dFd)_; *p* < 0.0001		
Parameter	DG region	CA1 region	CA3 region
**Astrocyte morphology**
Soma density	*F*_(2,84)_ = 30.53*	*F*_(2,84)_ = 22.83*	*F*_(2,84)_ = 24.37*
Soma area	*F*_(2,84)_ = 18.27*	*F*_(2,84)_ = 24.6*	*F*_(2,84)_ = 19.5*
Sum inters	*F*_(2,84)_ = 14.53*	*F*_(2,84)_ = 13.03*	*F*_(2,84)_ = 10.18*
Mean inters	*F*_(2,84)_ = 20.42*	*F*_(2,84)_ = 13.4*	*F*_(2,84)_ = 12.76*
Ramification index	*F*_(2,84)_ = 14.24*	*F*_(2,84)_ = 11.37*	*F*_(2,84)_ = 16.81*
Astrocyte length	*F*_(2,84)_ = 20.14*	*F*_(2,84)_ = 28.86*	*F*_(2,84)_ = 23.1*
**Microglia morphology**
Soma density	*F*_(2,84)_ = 9.216*	*F*_(2,84)_ = 54.27*	*F*_(2,84)_ = 25.22*
Soma area	*F*_(2,84)_ = 18.24*	*F*_(2,84)_ = 47.28*	*F*_(2,84)_ = 27.67*
Sum inters	*F*_(2,84)_ = 17.21*	*F*_(2,84)_ = 30.28*	*F*_(2,84)_ = 30.85*
Mean inters	*F*_(2,84)_ = 21.58*	*F*_(2,84)_ = 45.18*	*F*_(2,84)_ = 18.76*
Ramification index	*F*_(2,84)_ = 62.23*	*F*_(2,84)_ = 12.55*	*F*_(2,84)_ = 27.44*
Microglial length	*F*_(2,84)_ = 19.53*	*F*_(2,84)_ = 32.63*	*F*_(2,84)_ = 34.45*

Two way ANOVA followed by Tukey post hoc test with multiple comparison was applied for statistical comparison. *p < 0.05 is significantly different compared to control.

### Caffeine or Modafinil Treatment Maintained the Microglial Cells Numbers during SD

**Figure 7:**
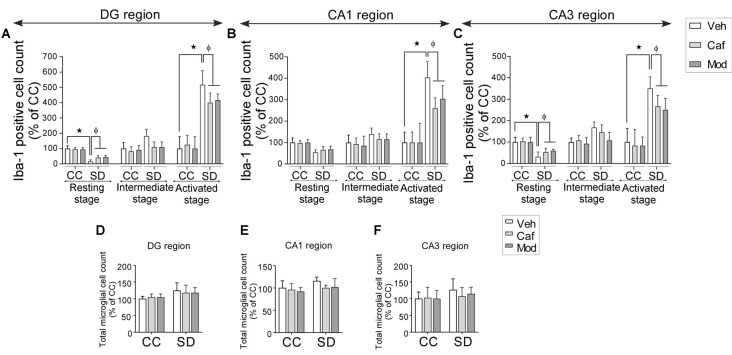
Caffeine or modafinil improved the microglial cell numbers following SD. Changes in the microglial cell count at different stages (resting, intermediate and activated) in **(A)** DG region; **(B)** CA1 region; **(C)** CA3 region of the hippocampus. Total microglial cell count in **(D)** DG region; **(E)** CA1 region; **(F)** CA3 region of the hippocampus. *p < 0.05 when compared to control treated with vehicle; ^φ^*p* < 0.05 when compared to sleep deprived treated with vehicle. Two way ANOVA followed by Tukey *post hoc* test with multiple comparison was applied for statistical comparison between groups and for the graphical representation, values expressed mean percentage of Control ± SEM.

We found a significant decrease in resting stage microglial cell in DG and CA3 region of the hippocampus, while the activated microglial cell numbers were significantly increased in DG, CA1 and CA3 region of the hippocampus. No significant change was observed in the intermediate state microglia cell count during SD. Caffeine or modafinil treatment during SD significantly increased the resting stage microglial cell and decreased activated microglial cell count, given during SD in DG (resting (*F*_(2,84)_ = 22.65; *p* < 0.0001; Boia et al., 2017); intermediate (*F*_(2,84)_ = 0.1197; *p* = 0.8874); activated (*F*_(2,84)_ = 33.82; *p* < 0.0001); Figure 7A), CA1 (resting (*F*_(2,84)_ = 4.741; *p* = 0.0112); intermediate (*F*_(2,84)_ = 0.03004; *p* = 0.9704); activated (*F*_(2,84)_ = 44.26; *p* < 0.0001); Figure 7B), and CA3 (resting (*F*_(2,84)_ = 11.15; *p* < 0.0001); intermediate (*F*_(2,84)_ = 0.09492; *p* = 0.9095); activated (*F*_(2,30)_ = 3.636; *p* < 0.0001; Figure 7C).

Although, trivial increase in total microglial cell count in SD-vehicle group was observed, caffeine or modafinil treatment also showed in consequential improvement in DG (*F*_(2,84)_ = 0.1095; *p* = 0.8964; Figure 7D); CA1 (*F*_(2,84)_ = 0.5845; *p* = 0.5597; Figure 7E), and CA3 (*F*_(2,84)_ = 0.8691; *p* = 0.4231; Figure 7F) region of the hippocampus.

### Changes in Mood Was Correlated to Microglial Activation Induced Up-regulated Level of Pro-inflammatory Cytokines during SD

The interaction between the anxiety parameters, pro-inflammatory cytokines and activated microglia cell count was evaluated by the correlation analysis to validate the findings.

**Figure 8:**
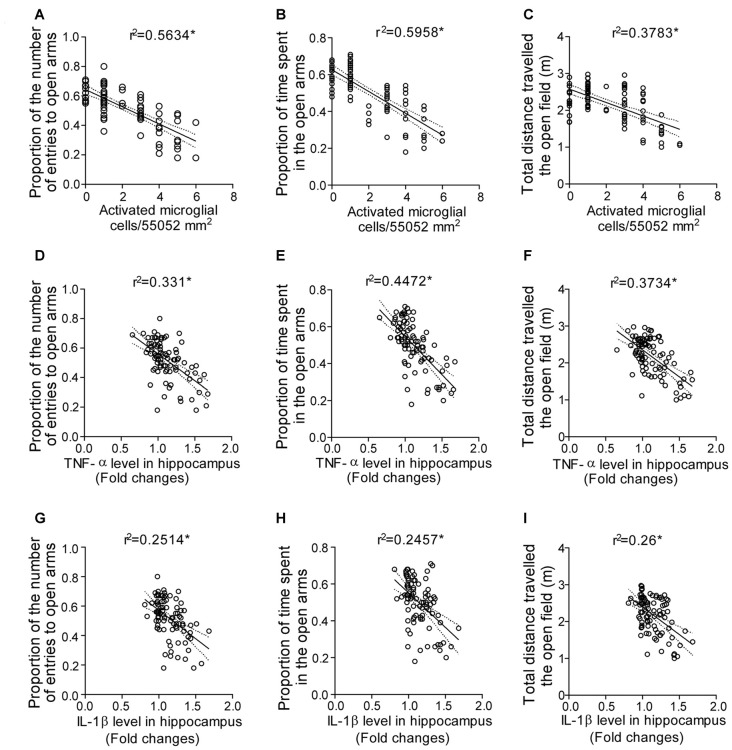
Correlation analysis predicting the interaction between the anxious behavior, pro-inflammatory cytokines and activated microglia cell during SD. Interaction between the anxious behavior and activated microglial cell as shown by correlation between **(A)** proportion of the number of entries in the open arms and activated microglial cell count in the hippocampus; **(B)** proportion of the time spent in the open arms and activated microglial cell count in the hippocampus; **(C)** total distance traveled in the OF and activated microglial cell count in the hippocampus. Finally, the interaction between the anxious behavior and pro-inflammatory cytokine levels as shown by correlation between **(D)** proportion of the number of entries in the open arms and TNF-α level in hippocampus; **(E)** proportion of the time spent in the open arms and TNF-α level in hippocampus; **(F)** total distance traveled in the OF and TNF-α level in hippocampus; **(G)** proportion of the number of entries in the open arms and IL-1β level in hippocampus; **(H)** proportion of the time spent in the open arms and IL-1β level in hippocampus; **(I)** total distance traveled in the OF and IL-1β level in hippocampus. Pearson’s test was applied for correlation analysis. *p* < 0.05 was considered to be statistically significant.

There was a significant correlation between the proportion of the number of entries in the open arms and the number of activated microglial cell count (*r*^2^ = 0.5634; *p* < 0.0001; Figure 8A); proportion of the time spent in the open arms and the number of activated microglial cell count (*r*^2^ = 0.5958; *p* < 0.0001; Figure 8B), and the total distance traveled in the OF and the number of activated microglial cell count (*r*^2^ = 0.3979; *p* < 0.0001; Figure 8C).

A significant correlation was observed between the proportion of the number of entries in the open arms and TNF-α level in hippocampus (*r*^2^ = 0.331; *p* < 0.0001; Figure 8D); proportion of the time spent in the open arms and TNF-α level in hippocampus (*r*^2^ = 0.4472; *p* < 0.0001; Figure 8E), and the total distance traveled in the OF and TNF-α level in hippocampus (*r*^2^ = 0.3734; *p* < 0.0001; Figure 8F). Similarly, correlation analysis showed significant correlation between the proportion of the number of entries in the open arms and IL-1β level in hippocampus (*r*^2^ = 0.2514; *p* < 0.0001; Figure 8G); proportion of the time spent in the open arms and IL-1β level in hippocampus (*r*^2^ = 0.2457; *p* < 0.0001; Figure 8H) and the total distance traveled in the OF and IL-1β level in hippocampus (*r*^2^ = 0.26; *p* < 0.0001; Figure 8I).

## Discussion

SD affected depressive/anxiety-like behavior and neuroinflammatory reactivity. Sleep deprived rats were more anxious in the EPM and OF test. However, caffeine or modafinil treatment increased OF activity, and thus reversed the effects of SD. Moreover, caffeine or modafinil decreased the time in closed but increased the time in open arms. Finally, pro-inflammatory cytokine levels were increased following SD and caffeine/modafinil decreased the pro-inflammatory and increased anti-inflammatory cytokine levels. As anticipated, the hippocampus of SD rats had higher glial immunoreactivity and altered morphology, and this effect was more pronounced in the microglial cells number, while psychostimulant drugs improved the above consequences. However, the tested doses of caffeine were higher and did not mimic habitual caffeine consumption. Together, our data highlight the influence of SD on neuroinflammatory responsiveness, and the importance of considering these factors in animal tests of depression/anxiety behaviors.

### Caffeine or Modafinil Treatment Improved the Physiological Consequences during SD

Normal sleep is necessary for health and sleep disruption influences the physiological function (Kumar and Kalonia, 2007). Food intake and mood are related to each other, depending on the stress, food intake may decrease or increase (Singh, 2014). There is a controversial relationship between the body weight, food intake and sleep. There are reports on body weight reduction during SD (Mavanji et al., 2013), weight gain attenuation with no change in food intake during SD (Barf et al., 2012), at the same time increase in food intake with decreases in body weight (Koban and Stewart, 2006). Animal studies had shown that increased cytokine levels reduce food intake and body weight during neuroinflammatory conditions (Park et al., 2011a; Zombeck et al., 2013). In contrast to previous studies, we observed a decrease in body weight of sleep-deprived rats, which was improved after caffeine/modafinil treatment.

### Caffeine or Modafinil Administration Produced Anxiolytic Effect and Recovered from the Depressive Behavior during SD

Under stressful conditions, the anxiety index has been found to be increased (Pechlivanova et al., 2012). Additionally, there are various reports of anxiogenic behavior in rodents as shown by EPM (decrease in time spent in open arms) and OF (impaired locomotor activity) test with body weight loss during SD (Silva et al., 2004; Garcia et al., 2011; Alkadhi et al., 2013; Matzner et al., 2013). Caffeine has a dose-dependent effect on anxious behavior. The previous findings revealed the behavioral observations that low (10 mg/kg) or moderate (20 mg/kg) dose of caffeine administration had shown reduction in anxiety behavior revealed by increased locomotor activity (Poletaeva and Oleinik, 1975; Antoniou et al., 1998), while increased dose of caffeine showed anxiogenic effect (Kayir and Uzbay, 2006) as predicted by the decrease or increase time and entry in open arms in rodents. Caffeine had shown to decrease the anxiety level during bright light stress conditions (Hughes et al., 2014) and chronic unpredictable stress conditions in rodents (Kaster et al., 2015). Like rodent studies, caffeine administration showed mood improvement in humans also (Smith et al., 2006). There are also reports available showing no effects of caffeine on anxiety (Khor et al., 2013). Similar to caffeine, modafinil showed anxiolytic effect shown by increased locomotor activity in humans (van Vliet et al., 2006) as well as rodents (Siwak et al., 2000; Quisenberry et al., 2013). This data is in consistent with the previous reports showing anxiogenic behavior during SD and caffeine or modafinil treatment improved the anxiety state during SD in rats, although the exact mechanism is still unclear.

There is a close association of the anxiety and depression with the disturbance in the normal sleep-wake cycle (Grønli et al., 2004; Jakubcakova et al., 2012; Kostyalik et al., 2014), immune system activation, assessed by decreased preference for a sweetness increased immobility time (Park et al., 2011b; Jangra et al., 2014). Imbalance in the cytokines level upon immune system activation has been found to be responsible for depression, predicted by a decrease in sucrose preference and increase in immobility (Ballok and Sakic, 2008; Braun et al., 2012). Inflammatory stimulation such as lipopolysaccharide model (Sayd et al., 2014) and Poly I:C administration (Missault et al., 2014), increased the pro-inflammatory cytokine (IL-1β, TNF-α and IL-6) levels and induced depressive symptoms. Furthermore, sickness behavior had also been found to be associated with the increased levels of the pro-inflammatory cytokine (Bluthé et al., 1995; Konsman et al., 2002; Dantzer, 2004; Vichaya et al., 2016, 2017). The incidence of anhedonia (decreased intake of sweet solution) had been reported in humans after SD (Petrovsky et al., 2014). Previous studies indicate a positive effect of caffeine on depressive symptoms shown by reduced immobility in rats (Vieira et al., 2008; Rusconi et al., 2014). Caffeine administration had been reported to alleviate the depressive behavior and memory dysfunction during chronic stress in a study by Kaster group (Kaster et al., 2015). Furthermore, inability in the reversal of the mood deficits in helpless mice during caffeine intake was reported (Machado et al., 2017). Human studies had shown improvement after modafinil treatment given during depression (Price and Taylor, 2005; Frye et al., 2007). Similar to humans, modafinil showed improvement in body weight along with mobility during stress in rats (Regenthal et al., 2009). In the paradigm used for our study, SD rats showed an increase in immobility and decrease the preference for sucrose solution, but the changes observed were not significant. Our data revealed non-significant decrease in immobility and increased sucrose preference after caffeine/modafinil treatment during SD.

### Caffeine or Modafinil Administration Maintained the Inflammatory Profile during SD

The increase in the cytokine levels with decreased food and water intake had been reported in neuroinflammatory models of rodents (Zombeck et al., 2013). The anxiogenic and depressive effect of cytokines shown by a preference for closed arms than open arms reduced immobility and decreased sucrose preference validated by knock-out and cytokines administration studies (Pan et al., 2013). One rodent study on maternal obesity reported an elevated level of pro-inflammatory cytokines along with mood disorder (Kang et al., 2014); another neuroinflammatory study on lupus-prone mice described anxious behavior along with an increased pro-inflammatory cytokine level (Ballok and Sakic, 2008). The previous report suggested the beneficial effects of caffeine on MDMA induced a behavioral and neuroinflammatory response (Ruiz-Medina et al., 2013). Caffeine administration showed the ability to control the behavioral alterations in neuroinflammatory disease models such as Parkinson’s (Chen et al., 2001; Joghataie et al., 2004) and Alzheimer’s (Arendash et al., 2006; Dall’Igna et al., 2007). Caffeine treatment also restored the memory performance and glial cells reactivity in a rodent model of diabetes (Duarte et al., 2012) and Machado-Joseph disease (Gonçalves et al., 2013). Our data showed the increased level of pro-inflammatory and decreased levels of anti-inflammatory cytokine in hippocampus and plasma during SD. Caffeine or modafinil treatment improved the cytokine levels in the hippocampus and plasma during SD.

Astrocytes, immunocompetent cells of the brain, becomes activated and secretes several neurotoxic substances along with an enhanced GFAP protein expression (astrogliosis protein marker). This enhanced GFAP expression relates to the astrocytes activation severity. Astrocytes activation assessed by GFAP expression was increased along with increased cytokine levels during inflammatory stimulation by LPS administration (Brahmachari et al., 2006; Park et al., 2011a; Norden et al., 2016). Microglial activation marker (Iba-1) showed increased expression associated with anxiety in maternal obesity model (Kang et al., 2014), pollutants exposure (Bolton et al., 2012) in rodents. The association between the mood alterations and glial cells reactivity under the perspective of the purinergic neuromodulation had been recently studied (Rial et al., 2016). Caffeine decreases the glial cells activation along with the reducing production of pro-inflammatory cytokines due to the localization of the adenosine receptors on microglia (Sonsalla et al., 2012). In rodents, caffeine treatment has been reported to decrease the astrocytes activity (Ardais et al., 2014) and microglial activation during rodent models of neuroinflammation such as maneb and paraquat (Yadav et al., 2012) and MDMA (Ruiz-Medina et al., 2013). Our results also highlight the influence of SD on the immunoreactivity of astrocytes and microglia (increased) and caffeine or modafinil treatment down-regulated the immunoreactivity of astrocytes and microglia during SD.

We also observed the morphological changes in the astrocytes and microglia in the hippocampus of sleep-deprived rats and caffeine or modafinil administration during SD improved these changes during SD. Results of this study get supported from the previous studies of social defeat, showing elevated cytokine levels in the brain associated with anxiety (Wohleb et al., 2014a, b). Along with morphological changes, we observed increased cell numbers of activated microglial cells and decreased cell count of resting stage microglial cell during SD, while caffeine or modafinil treatment following SD improved the resting and activated microglial cells count. Previous findings dictated the modulatory role of adenosine A2A receptor system in neuroprotection by controlling the microglia inducing neuroinflammation (Dai et al., 2010; Rebola et al., 2011; Gyoneva et al., 2014b). In a previous study on stroke model, increased activated microglial cells, morphological alteration of microglia along with anxiety and depression-like behavior was reported (Nemeth et al., 2014). A recent study enlightened an interplay between the microglial cells alterations and anxiety disorders, regulated by adenosine A2A receptor (Caetano et al., 2017). Stressful conditions trigger the increased release of adenosine and ATP, in which the adenosine modulation system in association with glial cells afford maximum neuroprotection (Cunha, 2016). Caffeine attenuated the activated microglial cell count in the hippocampus on LPS stimulation (Brothers et al., 2010) and high cholesterol diet-induced model of neuroinflammation (Chen et al., 2008). Caffeine administration prevented the microglia activation induced neuroinflammation in the transient retinal ischemic model in rodents (Boia et al., 2017). Similarly, modafinil treatment prevented the glial cells activation shown by increased resting and decrease activated stage cell with improvement in immunoreactivity in a neuroinflammatory model in rodents (Raineri et al., 2012). It was observed that microglial cells activation induced the increased level of pro-inflammatory cytokines, which further influenced the normal mood of rats towards anxious state during SD. The use of only male Sprague Dawley rats is a limitation of the present study as recent survey suggested the relation of sleep patterns with caffeine is different in males and females (Frozi et al., 2018).

## Conclusion

The present study demonstrated that inhibition of microglial cells by caffeine or modafinil treatment modulated the cytokine levels with increased the anti-inflammatory cytokines and ameliorates the anxious behavior) during SD. Our data suggested that caffeine/modafinil are the effective therapeutic agents against SD-induced neuroinflammation and anxiety behavior.

## Author Contributions

UP and MW designed the study and wrote the manuscript. MW performed the experiments and analyzed the data. KRay, LT, GC, KRoy, SS, SD and VJ helped in the manuscript writing. GC, KRoy and VJ helped in the immunohistochemistry of glial proteins and cytokines level measurement. SS and SD helped in the behavioral and RT-PCR experiments. UP, KRay, KK and LT contributed in procurement and facilitated the instruments and other facilities. All authors read and approved the final manuscript.

## Conflict of Interest Statement

The authors declare that the research was conducted in the absence of any commercial or financial relationships that could be construed as a potential conflict of interest.

## Supplementary Material

FIGURE S1 Caffeine or modafinil treatment improved the sleep deprivation (SD)-induced physiological changes in rats. Changes in **(A)** body weight; **(B)** food intake. **p* < 0.05 when compared to control treated with vehicle; ^φ^*p* < 0.05, when compared to sleep deprived treated with vehicle. Two way ANOVA followed by Tukey *post hoc* test with multiple comparison was applied for statistical comparison between groups and for the graphical representation, values expressed mean percentage of Control ± SEM.

FIGURE S2 Fold changes in the inflammatory cytokines in plasma during caffeine/modafinil administration following SD. Fold changes in **(A)** TNF-α; **(B)** IL-1β; **(C)** IL-6 (pro-inflammatory cytokines); **(D)** IL-4; **(E)** IL-10 (anti-inflammatory cytokines) in plasma. **p* < 0.05 when compared to control treated with vehicle; ^φ^*p* < 0.05 when compared to sleep deprived treated with vehicle. Two way ANOVA followed by Tukey *post hoc* test with multiple comparison was applied for statistical analysis.

## Abbreviations

DAB: Diaminobenzidine
ELISA: Enzyme-linked immunosorbent assay
EPM: Elevated plus maze
FST: Forced swim test
GFAP: Glial fibrillary acidic protein
Iba-1: Ionized calcium-binding adapter molecule I
OF: Open field
PBS: Phosphate buffered saline
PFA: Paraformaldehyde
PBST: Phosphate buffered saline containing tween-20/tritonX-100
SD: Sleep deprivation.
