# Correction: Caffeine and modafinil ameliorate the neuroinflammation and anxious behavior in rats during sleep deprivation by inhibiting the microglia activation

**DOI:** 10.3389/fncel.2026.2024052

**Published:** 2026-10-30

**Authors:** Meetu Wadhwa, Garima Chauhan, Koustav Roy, Surajit Sahu, Satyanarayan Deep, Vishal Jain, Krishna Kishore, Koushik Ray, Lalan Thakur, Usha Panjwani

**Affiliations:** Defence Institute of Physiology & Allied Sciences (DIPAS), Defence Research and Development Organization (DRDO), New Delhi, India

**Keywords:** sleep deprivation, mood changes, microglia, cytokines, neuroinflammation, caffeine, modafinil

There was a mistake in Figures 2B, 2C, and 2E as published. Few technical replicates were included during initial analysis which causes pseudorandomisation of data. The corrected Figure 2 appears below.

**Figure 2:**
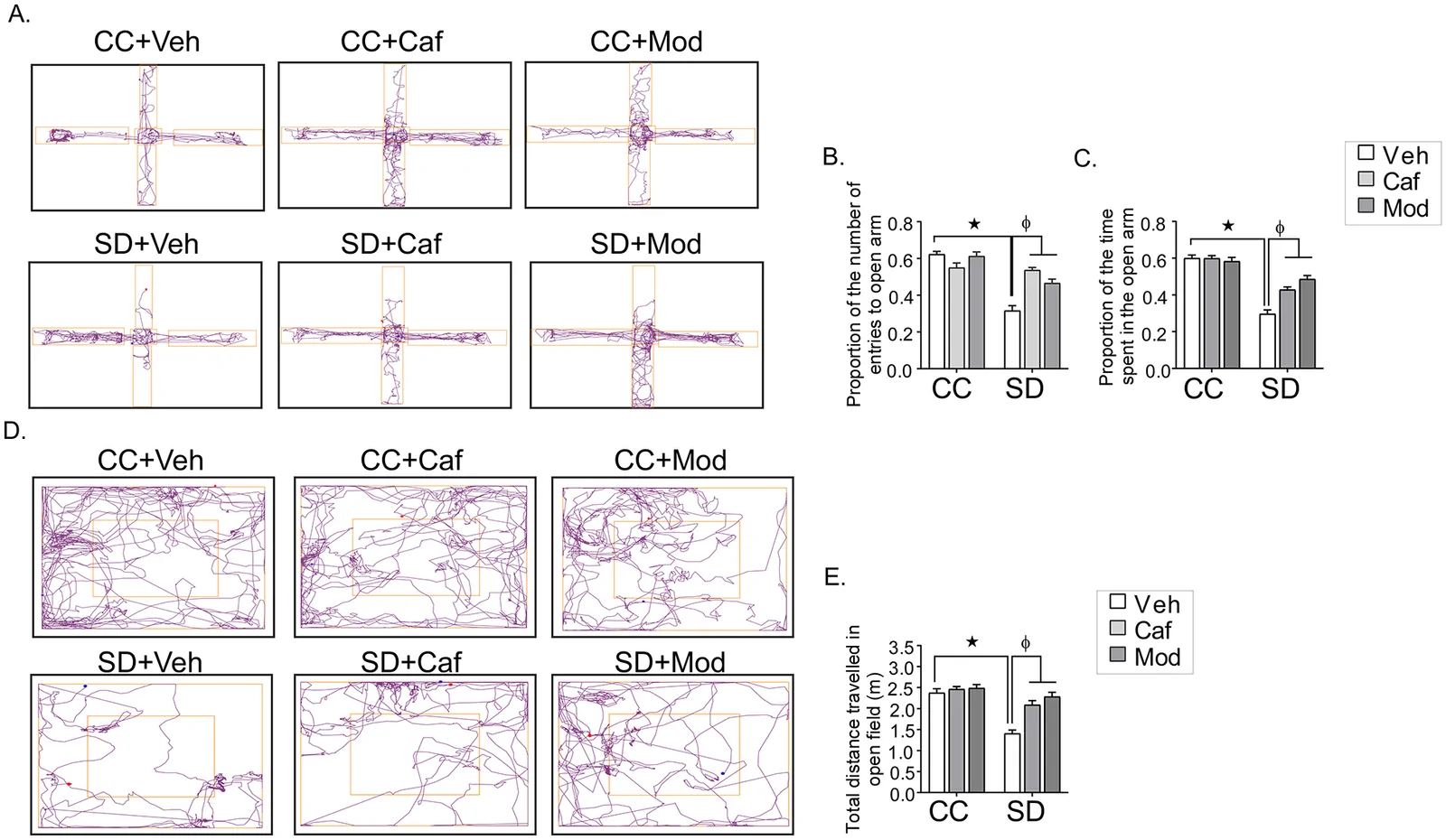
Assessment of the anxious behavior following caffeine/modafinil treatment during sleep deprivation (SD). **(A)** Track plot of the rats during elevated plus maze (EPM) test and the study parameters: **(B)** proportion of the number of entries in the open arms; **(C)** proportion of the time spent in the open arms. **(D)** Track plot of the rats during open field (OF) test; **(E)** total distance traveled in the OF. *p < 0.05 when compared to control treated with vehicle; ^ϕ^*p* < 0.05 when compared to sleep deprived treated with vehicle. Two way ANOVA followed by Tukey *post hoc* test with multiple comparison was used.

There was a mistake in the caption of **Figure 5A** as published. This sentence should be as - Information related to partial overlap among (CC+Veh-Caf-Mod-GFAP-DG) and (CC+Veh-Caf-Mod-GFAP-CA1) was missing in the original article. The corrected caption of **Figure 5A** appears below.

**Figure 5 (A)** Representable image of astrocytes expression in DG, CA1, and CA3 regions of the hippocampus. For each experimental group, images of the DG, CA1, and CA3 regions were acquired from the same animal to maintain within-group consistency. All hippocampal subregions were imaged at 10× magnification. Given the close anatomical proximity of these regions and the field of view at this magnification, partial inclusion of the upper DG region in images centered on the lower CA1 region may occur and is difficult to avoid.

A correction has been made to the section [**Results**, *Caffeine/modafinil administration produced anxiolytic effect during SD*, paragraph 1 and 2]:

“An incorrect statistical value was provided for the proportion of the number of entries [*F*_(2, 114)_ = 17.4; *p* < 0.0001; Figure 2B], the proportion of the time spent in the open arms test [*F*_(2, 114)_ = 25.19; *p* < 0.0001; Figure 2C], and the total distance traveled in the open frame [*F*_(2, 114)_ = 14.91; *p* = 0.0001; Figure 2E]. The correct values are [*F*_(2, 84)_ = 23.54; *p* < 0.0001) for the proportion of the number of entries (Figure 2B), [*F*_(2, 84)_ = 17.14; *p* < 0.0001] for the proportion of the time spent in the open arms (Figure 2C), and [*F*_(2, 84)_ = 10.14; *p* < 0.0001] for the total distance traveled in the open frame (Figure 2E).”

A correction has been made to the section [**Materials and methods**, *Imaging and analysis*, paragraph 1]:

“An additional information is added for better explanation of possible overlap in the DG and CA1 region images. The correct paragraph is “The immune-stained sections were observed under Olympus (Melville, NY, USA) BX51TF microscope and images were taken from the DG, CA1 and CA3 regions of the dorsal hippocampus of the brain. We aimed to represent the distinct hippocampal regions (DG, CA1, and CA3) at their respective anatomical locations across the different experimental groups. For each group, images of the DG, CA1, and CA3 regions were obtained from the same animal to maintain consistency within that group. All images were captured at 10 × magnification. Due to this magnification and the close anatomical proximity of the hippocampal subregions, a small portion of the upper DG region may be visible in images focused on the CA1 region. This minor overlap is technically difficult to avoid at this magnification. We performed sholl analysis for the morphological evaluation of astrocytes and microglial cells. The cell quantification was performed using stereo investigator program. Similarly, immunoreactivity of astrocytes and microglial cells was quantified with the help of ImageJ software.””

The original version of this article has been updated.

