# Supplementary figures and images for: Caffeine and Modafinil Ameliorate the Neuroinflammation and Anxious Behavior in Rats during Sleep Deprivation by Inhibiting the Microglia Activation
Source: Front Cell Neurosci. 2018 Feb 28;12:49. doi: 10.3389/fncel.2018.00049 (PMC5863523; doi:10.3389/fncel.2018.00049)

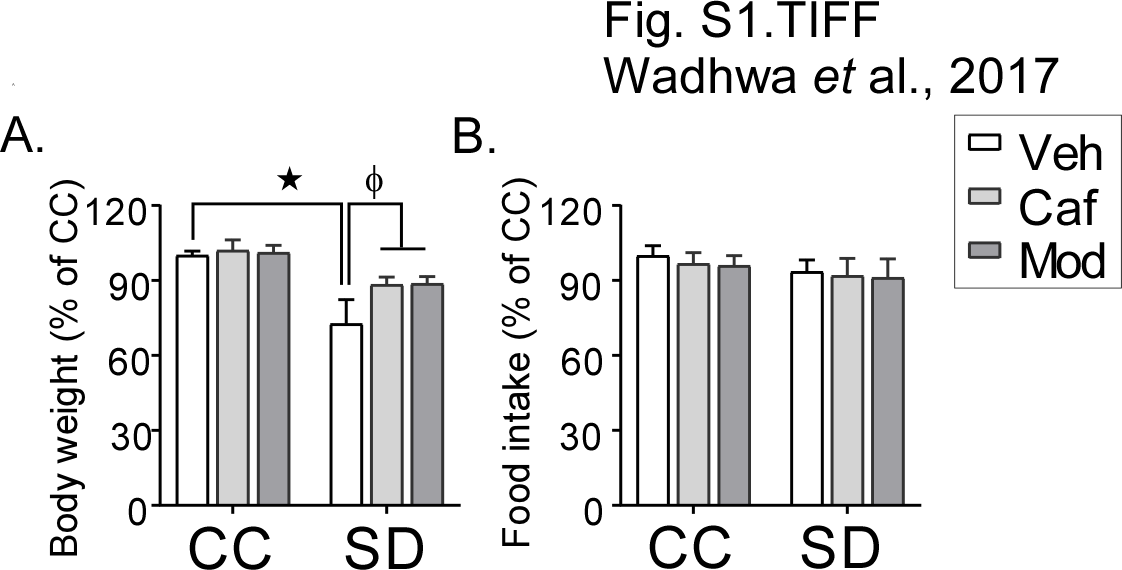

Supplement: FIGURE S1 — Caffeine or modafinil treatment improved the sleep deprivation (SD)-induced physiological changes in rats. Changes in (A) body weight; (B) food intake. *p < 0.05 when compared to control treated with vehicle; φp < 0.05, when compared to sleep deprived treated with vehicle. Two way ANOVA followed by Tukey post hoc test with multiple comparison was applied for statistical comparison between groups and for the graphical representation, values expressed mean percentage of Control ± SEM. [file Image_1.tif]

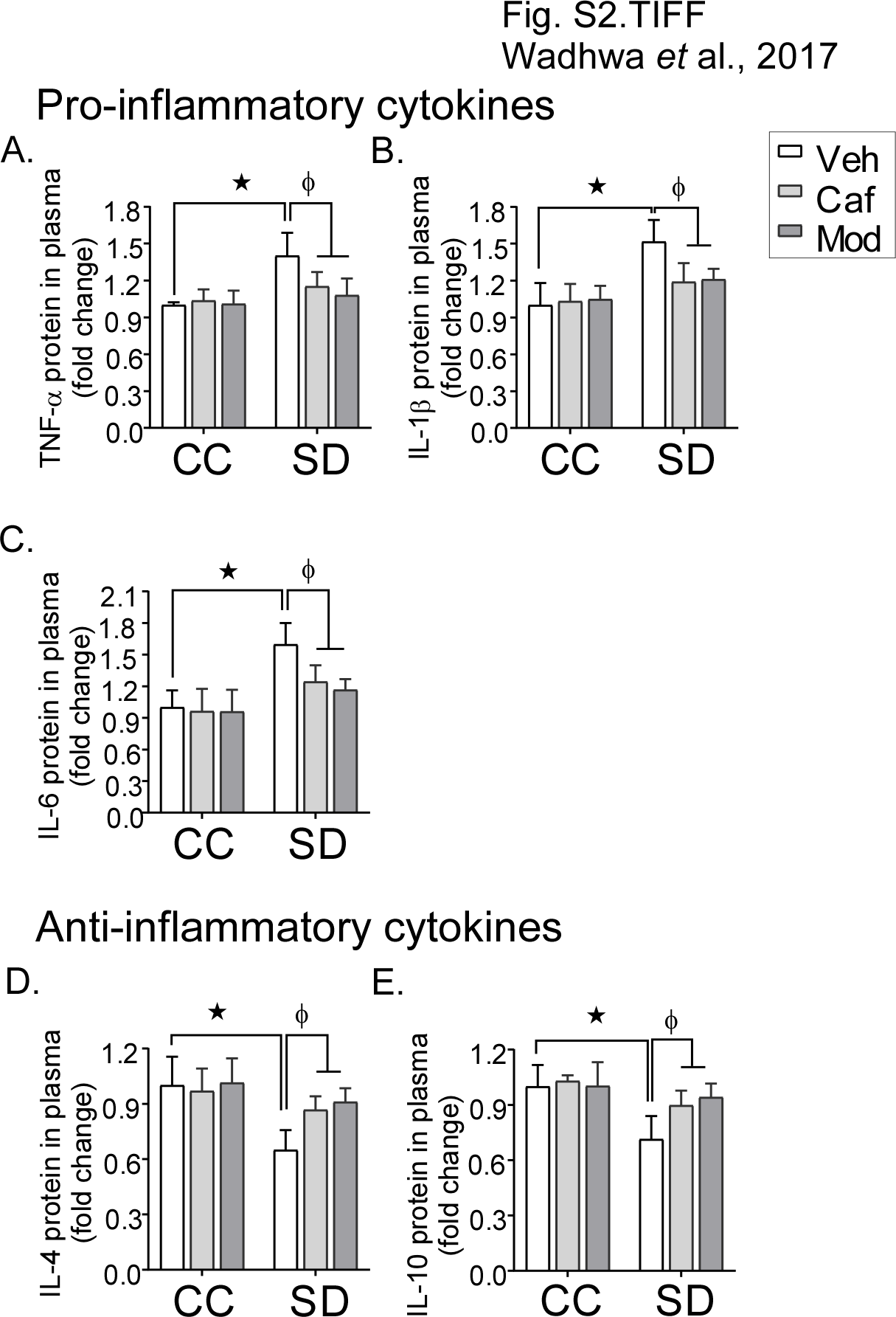

Supplement: FIGURE S2 — Fold changes in the inflammatory cytokines in plasma during caffeine/modafinil administration following SD. Fold changes in (A) TNF-α; (B) IL-1β; (C) IL-6 (pro-inflammatory cytokines); (D) IL-4; (E) IL-10 (anti-inflammatory cytokines) in plasma. *p < 0.05 when compared to control treated with vehicle; φp < 0.05 when compared to sleep deprived treated with vehicle. Two way ANOVA followed by Tukey post hoc test with multiple comparison was applied for statistical analysis. [file Image_2.tif]
